# Novel methods for coupled prediction of extreme wind speeds and wave heights

**DOI:** 10.1038/s41598-023-28136-8

**Published:** 2023-01-20

**Authors:** Oleg Gaidai, Yihan Xing, Xiaosen Xu

**Affiliations:** 1 Shanghai Ocean University, Shanghai, China; 2 University of Stavanger, Stavanger, Norway; 3 Jiangsu University of Science and Technology, Zhenjiang, China

**Keywords:** Climate sciences, Environmental sciences, Natural hazards, Engineering, Physics

## Abstract

Two novel methods are being outlined that, when combined, can be used for spatiotemporal analysis of wind speeds and wave heights, thus contributing to global climate studies. First, the authors provide a unique reliability approach that is especially suited for multi-dimensional structural and environmental dynamic system responses that have been numerically simulated or observed over a substantial time range, yielding representative ergodic time series. Next, this work introduces a novel deconvolution extrapolation technique applicable to a wide range of environmental and engineering applications. Classical reliability approaches cannot cope with dynamic systems with high dimensionality and responses with complicated cross-correlation. The combined study of wind speed and wave height is notoriously difficult, since they comprise a very complex, multi-dimensional, non-linear environmental system. Additionally, global warming is a significant element influencing ocean waves throughout the years. Furthermore, the environmental system reliability method is crucial for structures working in any particular region of interest and facing actual and often harsh weather conditions. This research demonstrates the effectiveness of our approach by applying it to the concurrent prediction of wind speeds and wave heights from NOAA buoys in the North Pacific. This study aims to evaluate the state-of-the-art approach that extracts essential information about the extreme responses from observed time histories.

## Introduction

It is challenging to accurately assess multi-dimensional dynamic system reliability using conventional theoretical reliability methods^1–4^. Estimating the reliability of a complicated dynamic system is easy if sufficient system response data are available or if direct Monte Carlo simulations are performed^5^. However, experimental or computational costs may be expensive for many engineering dynamic systems with complicated dynamics. Motivated by this, the authors developed a new reliability method for dynamic systems that require significantly less measurement and computing costs.

It has been a challenge to how exactly one predicts the shape and characteristics of waves through wind speed variables, even though this has been started and partially solved in the works of^6–11^. Our work, however, deals with the general approach of extreme value theory in which no physical dynamics of the water waves are expected to play a significant role in driving the appearance of rare events, i.e. there is an expected universality of extreme events in a variety of different physical and natural systems^12–17^. Although beyond the scope of our work, there Some successful attempts have been to study extreme events in water waves (often called rogue or freak waves) with distributions uniquely determined by the dynamics of the physical system. For instance^18,19^, have shown that water waves departing from linear theory will modify their distribution from a Rayleigh type^20–22^ to a distribution dependent on the square root of the wave steepness. Similarly^23,24^, have shown that a Rayleigh distribution modified by a polynomial function of the ratio between height and water depth controls extreme events in Hurricane data. In addition, spectrum bandwidth seems to have different types of effects in extreme wave distribution depending on whether they are in deep^25–28^ or shallow water^13^. Furthermore, ocean processes such as shoaling or wave-current systems that drive wave trains out of equilibrium have been experimentally^22,25,29–37^ associated with increasing the occurrence of extreme waves by order of magnitude. However, it has been recently found that no established theoretical distribution to date, neither universal as Gumbel nor based on physical principles, can describe extreme wave statistics in a wide range of conditions^6–37^.

Note that methods introduced by authors here do not rely on Gumbel (or any other type) distribution type assumption, instead, Gumbel-based extrapolation was used just for comparison.

## Method

Consider multi-degree of freedom (MDOF) jointly stationary dynamic environmental system with representative response vector process ${\varvec{R}}\left( t \right) = \left( {X\left( t \right), Y\left( t \right), Z\left( t \right), \ldots } \right)$, that has been either measured or simulated over a sufficiently long time span $\left( {0,T} \right)$. Unidimensional global maxima over entire time span $\left( {0,T} \right)$ is denoted as $X_{T}^{{{\text{max}}}} = \mathop {\max }\limits_{0 \le t \le T} X\left( t \right)$, $Y_{T}^{{{\text{max}}}} = \mathop {\max }\limits_{0 \le t \le T} Y\left( t \right)$, $Z_{T}^{{{\text{max}}}} = \mathop {\max }\limits_{0 \le t \le T} Z\left( t \right), \ldots$.

By sufficiently long time span $T$ authors mean large enough value of $T$ with respect to the environmental dynamic system auto-correlation time. Let $X_{1} , \ldots ,X_{{N_{X} }}$ be consequent temporal local maxima of the environmental process $X\left( t \right)$ at discrete monotonously temporally increasing times $t_{1}^{X} < \ldots < t_{{N_{X} }}^{X}$ within $\left( {0,T} \right)$. Similar definition follows for other MDOF environmental response components $Y\left( t \right), Z\left( t \right), \ldots$ with $Y_{1} , \ldots ,Y_{{N_{Y} }} ;$ $Z_{1} , \ldots ,Z_{{N_{Z} }}$ and so on. For simplicity, all ${\varvec{R}}\left( t \right)$ components, maxima are assumed to be non-negative. The target now is to accurately estimate environmental system failure probability, namely its probability of exceedance

1 $$1 - P = {\text{Prob}}(X_{T}^{{{\text{max}}}} > \eta_{X} \cup Y_{T}^{{{\text{max}}}} > \eta_{Y} \cup Z_{T}^{{{\text{max}}}} > \eta_{Z} \cup \ldots )$$

where $P = \iiint {_{{\left( {0, 0, 0, , \ldots } \right)}}^{{\left( {\eta_{X} , \eta_{Y} , \eta_{Z } , \ldots } \right)}} }p_{{X_{T}^{{{\text{max}}}} , Y_{T}^{{{\text{max}}}} , Z_{T}^{{{\text{max}}}} , \ldots }} \left( {X_{T}^{{{\text{max}}}} , Y_{T}^{{{\text{max}}}} , Z_{T}^{{{\text{max}}}} , \ldots } \right)dX_{T}^{{{\text{max}}}} dY_{{N_{Y} }}^{{{\text{max}}}} dZ_{{N_{z} }}^{{{\text{max}}}} \ldots$ being probability of non-exceedance for critical values of environmental response components $\eta_{X}$, $\eta_{Y}$, $\eta_{Z}$,…; $\cup$ denotes logical unity operation «or»; $p_{{X_{T}^{{{\text{max}}}} , Y_{T}^{{{\text{max}}}} , Z_{T}^{{{\text{max}}}} , \ldots }}$ being joint probability density of the global maxima over the entire time span $\left( {0,T} \right)$.In practice, however, it is not always feasible to estimate directly $p_{{X_{T}^{{{\text{max}}}} , Y_{T}^{{{\text{max}}}} , Z_{T}^{{{\text{max}}}} , \ldots }}$ joint probability distribution due to its high dimensionality and limitations of the available data set. The moment when either $X\left( t \right)$ exceeds $\eta_{X}$, or $Y\left( t \right)$ exceeds $\eta_{Y}$, or $Z\left( t \right)$ exceeds $\eta_{Z}$, and so on, the environmental dynamic system being regarded as failed. Fixed failure levels $\eta_{X}$, $\eta_{Y}$, $\eta_{Z}$,… being individual for each unidimensional response component of ${\varvec{R}}\left( t \right)$. $X_{{N_{X} }}^{{{\text{max}}}} = {\text{max }}\{ X_{j} \,;j = 1, \ldots ,N_{X} \} = X_{T}^{{{\text{max}}}}$, $Y_{{N_{Y} }}^{{{\text{max}}}} = {\text{max }}\{ Y_{j} \,;j = 1, \ldots ,N_{Y} \} = Y_{T}^{{{\text{max}}}}$,$Z_{{N_{z} }}^{{{\text{max}}}} = {\text{max }}\{ Z_{j} \,;j = 1, \ldots ,N_{Z} \} = Z_{T}^{{{\text{max}}}}$, and so on.

Let sort local maxima time instants $\left[ {t_{1}^{X} < \ldots < t_{{N_{X} }}^{X} ;{ }t_{1}^{Y} < \ldots < t_{{N_{Y} }}^{Y} ;{ }t_{1}^{Z} < \ldots < t_{{N_{Z} }}^{Z} } \right]$ recorded in temporally non-decreasing order into one single synthetic merged time vector $t_{1} \le \ldots \le t_{N}$. Note that then $t_{N} = {\text{max }}\{ t_{{N_{X} }}^{X} , t_{{N_{Y} }}^{Y} , t_{{N_{Z} }}^{Z} , \ldots \}$, $N = N_{X} + N_{Y} + N_{Z} + \ldots$. In this case $t_{j}$ represents local maxima of one of MDOF environmental dynamic structural response components either $X\left( t \right)$ or $Y\left( t \right)$, or $Z\left( t \right)$ and so on. The latter means that having ${\varvec{R}}\left( t \right)$ time record, one only needs simultaneously and continuously screen for the system unidimensional response component local maxima and record its exceedance of MDOF limit vector $\left( { \eta_{X} ,{ }\eta_{Y} ,{ }\eta_{Z} ,...} \right)$ in any of dynamic system components $X, Y, Z, \ldots$ Local unidimensional response component maxima then merged into one synthetic temporal non-decreasing vector $\vec{R} = \left( {R_{1} , R_{2} , \ldots ,R_{N} } \right)$ in accordance with merged time vector $t_{1} \le \ldots \le t_{N}$. That is to say each local maxima $R_{j}$ is in fact actual encountered local maxima corresponding to either $X\left( t \right)$ or $Y\left( t \right)$, or $Z\left( t \right)$ and so on. Finally the unified limit vector $\left( {\eta_{1} , \ldots ,\eta_{N} } \right)$ is introduced with each component $\eta_{j}$ is either $\eta_{X}$, $\eta_{Y}$ or $\eta_{Z}$ and so on, depending which of $X\left( t \right)$ or $Y\left( t \right)$, or $Z\left( t \right)$ etc. corresponding to the current local maxima with running index $j$.

Scaling parameter $0 < \lambda \le 1$ being now introduced to artificially simultaneously decrease limit values for all system response components, i.e. new MDOF limit vector $\left( { \eta_{X}^{\lambda } , \eta_{Y}^{\lambda } ,{ }\eta_{z}^{\lambda } ,...} \right)$ with $\eta_{X}^{\lambda } \equiv \lambda \cdot \eta_{X}$, $\equiv \lambda \cdot \eta_{Y}$, $\eta_{z}^{\lambda } \equiv \lambda \cdot \eta_{Z}$, … being introduced. The unified limit vector $\left( {\eta_{1}^{\lambda } , \ldots ,\eta_{N}^{\lambda } } \right)$ being introduced with each component $\eta_{j}^{\lambda }$ being equal to either $\eta_{X}^{\lambda }$, $\eta_{Y}^{\lambda }$ or $\eta_{z}^{\lambda }$ and so on The latter automatically defines the target probability $P\left( \lambda \right)$ as a function of $\lambda$, note that $P \equiv P\left( 1 \right)$ from Eq. (1). Non-exceedance probability $P\left( \lambda \right)$ can be now estimated as follows

2 $$\begin{aligned} P\left( \lambda \right) & { } = {\text{Prob}}\left\{ {R_{N} \le \eta_{N}^{\lambda } , \ldots ,R_{1} \le \eta_{1}^{\lambda } } \right\} \\ & = {\text{Prob}}\{ R_{N} \le \eta_{N}^{\lambda } {|} R_{N - 1} \le \eta_{N - 1}^{\lambda } , \ldots ,R_{1} \le \eta_{1}^{\lambda } \} \cdot {\text{Prob}}\left\{ {R_{N - 1} \le \eta_{N - 1}^{\lambda } , \ldots ,R_{1} \le \eta_{1}^{\lambda } } \right\} \\ & = \mathop \prod \limits_{j = 2}^{N} {\text{Prob}}\{ R_{j} \le \eta_{j}^{\lambda } | R_{j - 1} \le \eta_{1j - }^{\lambda } , \ldots ,R_{1} \le \eta_{1}^{\lambda } \} \cdot {\text{Prob}}\left( {R_{1} \le \eta_{1}^{\lambda } } \right) \\ \end{aligned}$$

In practice the dependence between neighbouring $R_{j}$ is not always negligible, thus following one-step (will be called conditioning level $k = 1$) memory approximation being introduced

3 $${\text{Prob}}\{ R_{j} \le \eta_{j}^{\lambda } | R_{j - 1} \le \eta_{j - 1}^{\lambda } , \ldots ,R_{1} \le \eta_{1}^{\lambda } \} \approx {\text{Prob}}\{ R_{j} \le \eta_{j}^{\lambda } | R_{j - 1} \le \eta_{j - 1}^{\lambda } \}$$

for $2 \le j \le N$ (will be called conditioning level $k = 2$). Approximation introduced by Eq. (3) may be further expressed as

4 $${\text{ Prob}}\{ R_{j} \le \eta_{j}^{\lambda } | R_{j - 1} \le \eta_{j - 1}^{\lambda } , \ldots ,R_{1} \le \eta_{1}^{\lambda } \} \approx {\text{Prob}}\{ R_{j} \le \eta_{j}^{\lambda } | R_{j - 1} \le \eta_{j - 1}^{\lambda } , R_{j - 2} \le \eta_{j - 2}^{\lambda } \}$$

where $3 \le j \le N$ (called here conditioning level $k = 3$), and so on^38^. Equation (4) presents series of subsequent refinements of statistical independence assumption. The latter type of approximations models statistical dependence effect between neighbouring maxima with an increased accuracy. Since original MDOF dynamic process ${\varvec{R}}\left( t \right)$ was assumed ergodic and therefore stationary, probability $p_{k} \left( \lambda \right): = {\text{Prob}}\{ R_{j} > \eta_{j}^{\lambda } {|} R_{j - 1} \le \eta_{j - 1}^{\lambda } , R_{j - k + 1} \le \eta_{j - k + 1}^{\lambda } \}$ for $j \ge k$ is independent of $j$ but only dependent on conditioning level $k$. Thus non-exceedance probability may be now approximated

5 $$P_{k} \left( \lambda \right) \approx {\text{exp }}( - N \cdot p_{k} \left( \lambda \right))\, , k \ge 1.$$

Note that Eq. (5) follows from Eq. (1) by neglecting ${\text{ Prob}}\left( {R_{1} \le \eta_{1}^{\lambda } } \right) \approx 1$, as design failure probability must of a small order of magnitude, and *N*>>*k*. Equation (5) is similar to mean up-crossing rate equation for probability of exceedance^39–51^. There is convergence with respect to the conditioning parameter $k$

6 $$P = \mathop {\lim }\limits_{k \to \infty } P_{k} \left( 1 \right); \quad p\left( \lambda \right) = \mathop {\lim }\limits_{k \to \infty } p_{k} \left( \lambda \right)$$

Note that Eq. (5) for $k = 1$ turns into classic non-exceedance probability relationship with corresponding mean up-crossing rate function

7 $$P\left( \lambda \right){ } \approx {\text{exp }}( - \nu^{ + } \left( \lambda \right)\,T); \quad \nu^{ + } \left( \lambda \right) = \mathop \smallint \limits_{0}^{\infty } \zeta p_{{R\dot{R}}} \left( {\lambda ,\zeta } \right)d\zeta$$

where $\nu^{ + } \left( \lambda \right)$ denotes the mean up-crossing rate of the response level $\lambda$ for the above assembled non-dimensional vector $R\left( t \right)$ assembled from scaled MDOF system response $\left( {\frac{X}{{\eta_{X} }}, \frac{Y}{{\eta_{Y} }}, \frac{Z}{{\eta_{Z} }}, \ldots } \right)$. The mean up-crossing rate is given by the Rice's formula given in Eq. (7) with $p_{{R\dot{R}}}$ being joint probability density for $\left( {R, \dot{R}} \right)$ with $\dot{R}$ being the time derivative $R^{\prime}\left( t \right)$ ^1^. Equation (7) relies on the Poisson assumption, that is up-crossing events of high $\lambda$ levels (in this paper it is $\lambda \ge 1$) may be assumed to be independent.

The proposed methodology may be used for nonstationary cases as well. Given environmental scattered diagram of $m = 1,..,M$ sea states, each environmental short-term sea state having probability $q_{m}$, so that $\mathop \sum \limits_{m = 1}^{M} q_{m} = 1$. Next, let one introduce the long-term equation

8 $$p_{k} \left( \lambda \right) \equiv \mathop \sum \limits_{m = 1}^{M} p_{k} \left( {\lambda ,m} \right)q_{m}$$

with $p_{k} \left( {\lambda ,m} \right)$ being the same function as in Eq. (6), but corresponding to a specific short-term sea state with number $m$. The above introduced $p_{k} \left( \lambda \right)$ as functions being often regular in the tail, specifically for values of ${ }\lambda$ approaching and exceeding $1$. More specifically, for $\lambda \ge \lambda_{0}$, the probability distribution tail behaves similar to ${\text{exp}}\left\{ { - \left( {a\lambda + b} \right)^{c} + d} \right\}$ with $a, b, c, d$ being suitably fitted constants for suitable tail cut-on $\lambda_{0}$ value. Therefore, one can write

9 $$p_{k} \left( \lambda \right) \approx {\text{exp}}\left\{ { - \left( {a_{k} \lambda + b_{k} } \right)^{{c_{k} }} + d_{k} } \right\}, \lambda \ge \lambda_{0}$$

By plotting ${\text{ln}}\left\{ {{\text{ln}}\left( {p_{k} \left( \lambda \right)} \right) - d_{k} } \right\}$ versus ${\text{ln}}\left( {a_{k} \lambda + b_{k} } \right)$, often nearly perfectly linear tail behaviour being observed. Optimal values of the parameters $a_{k} , b_{k} , c_{k} ,p_{k} ,q_{k}$ may be determined using a sequential quadratic programming (SQP) method incorporated in the NAG Numerical Library^52^.

Let us consider stationary stochastic process $X\left( t \right)$, being either simulated or measured over a certain time span $0 \le t \le T$, and which is being represented as a sum of two independent identically distributed stationary environmental dynamic processes $X_{1} \left( t \right)$ and $X_{2} \left( t \right)$, namely

10 $$X\left( t \right) = X_{1} \left( t \right) + X_{2} \left( t \right)$$

Denoting probability density function (PDF) of $X\left( t \right)$ as $p_{X}$ and PDF of $X_{1} \left( t \right)$ and $X_{2} \left( t \right)$ as $p_{{X_{1} }}$, following convolution equation holds

11 $$p_{X} = {\text{conv}}\left( { p_{{X_{1} }} , p_{{X_{1} }} } \right)$$

In order to exemplify the latter idea regarding how to estimate unknown probability distribution robustly $p_{{X_{1} }}$, subsequently improving given empirical distribution $p_{X} .$ In the next, authors briefly discuss common knowledge regarding the discrete convolution of two vectors. The convolution of two vectors, ${\varvec{u}}$ and ${\varvec{v}}$, representing an area of overlap of vector components, as ${\varvec{v}}$ slides across ${\varvec{u}}$. Algebraically, convolution being the same operation as multiplying polynomials whose coefficients are the elements of, ${\varvec{u}}$ and ${\varvec{v}}$. Let $m = {\text{length}}\left( {\varvec{u}} \right)$ and $n = {\text{length}}\left( {\varvec{v}} \right)$. Then ${\varvec{w}}$ being the vector of length $m + n - 1$, with $k$-th element being

12 $$w\left( k \right) = \mathop \sum \limits_{j = 1}^{m} u\left( j \right)v\left( {k - j + 1} \right)$$

The sum is over all the values of $j$ that lead to legal subscripts for $u\left( j \right)$ and $v\left( {k - j + 1} \right)$, specifically $j = {\text{max}}\left( {1,k + 1 - n} \right):1:{\text{min}}\left( {k,m} \right)$. When $m = n$, as will be the main case in this study

13 $$\begin{aligned} &w\left( 1 \right) = u\left( 1 \right) \cdot v\left( 1 \right) \\ &w\left( 2 \right) = u\left( 1 \right) \cdot v\left( 2 \right) + u\left( 2 \right) \cdot v\left( 1 \right) \\ &w\left( 3 \right) = u\left( 1 \right) \cdot v\left( 3 \right) + u\left( 2 \right) \cdot v\left( 2 \right) + u\left( 3 \right) \cdot v\left( 1 \right) \\ & \cdots \\ &w\left( n \right) = u\left( 1 \right) \cdot v\left( n \right) + u\left( 2 \right) \cdot v\left( {n - 1} \right) + \cdots + u\left( n \right) \cdot v\left( 1 \right) \\ & \cdots \\ &w\left( {2n - 1} \right) = u\left( n \right) \cdot v\left( n \right) \\ \end{aligned}$$

From Eq. (12) one can also observe that having found ${\varvec{u}} = {\varvec{v}} = \left( {u\left( 1 \right),..,u\left( n \right)} \right)$, one can gradually obtain ${\varvec{w}}$-components $w\left( {n + 1} \right), \ldots ,w\left( {2n - 1} \right)$, as index increases from $n + 1$ to $2n - 1$. The latter clearly would extend vector ${\varvec{w}}$ into support domain that is twice longer than the original distribution support domain, i.e. doubling the $p_{X}$ distribution support length $\left( {2n - 1} \right) \cdot \Delta x \approx 2n \cdot \Delta x = 2X_{L}$, as compared to the original probability distribution support length $n \cdot \Delta x = X_{L}$ with $\Delta x$ being constant length of each descrete distribution bin. Note that ${\varvec{w}} = \left( {w\left( 1 \right), \ldots ,w\left( n \right)} \right)$ being discrete representation of the target empirical distribution $p_{X}$, and $n$ representing length of probability distribution support $\left[ {0,X_{L} } \right]$, for simplicity in this paper one is limited to the case of one-sided positive valued random variables, namely $X \ge 0$. As will be further discussed, a simple linear extrapolation of self deconvoluted vector $\left( {u\left( 1 \right), \ldots ,u\left( n \right)} \right)$ towards $\left( {u\left( {n + 1} \right), \ldots ,u\left( {2n - 1} \right)} \right)$ is suggested, with $p_{{X_{1} }}$ having its tail linearly extrapolated within the range $\left( {X_{L} ,2X_{L} } \right).$ Using Eq. (11) original vector ${\varvec{w}}$ will be extended and extrapolated into support domain being twice longer than original distribution support domain, namely doubling $p_{X}$ distribution support length $\left( {2n - 1} \right) \cdot \Delta x \approx 2n \cdot \Delta x = 2X_{L}$, as compared to original probability distribution support length $n \cdot \Delta x = X_{L}$. To smoothen original distribution $p_{X} \left( x \right)$ tail, authors have performed distribution $p_{X}$ tail interpolation for high tail values $x$, using Naess-Gaidai (NG) extrapolation method^53^. As has been discussed above, the proposed deconvolution extrapolation technique has an advantage of not presuming any specific extrapolation functional class needed to extrapolate distribution tail.

To validate the above-suggested extrapolation methodology, the «shorter» version of the original data set was used for extrapolation to compare with predictions based on a full «longer» data set.

## Results

This section aims to demonstrate the efficiency of the previously described methodology by applying the new method to the National Oceanic and Atmospheric Administration buoy (NOAA) ten-minute average wind speed values and significant wave heights (calculated as the average of the highest one-third of all wave heights during the 20-min sampling period) in the North Pacific region near the Hawaiian Islands. For this study, the NOAA wind and wave measurement location Station 51003 (LLNR 28005.7)—WESTERN HAWAII—205 NM SW of Honolulu was selected, and its measured wind speed and wave height values were set as two environmental system components (dimensions) *X*, *Y*, thereby serving as an example of a two-dimensional (2D) environmental system. Unidimensional extreme response values were chosen as the whole analysed data set maximum wind speeds and wave heights respectively during observation period between years 2009–2014 for the chosen buoy in situ measurement location.

**Figure 1:**
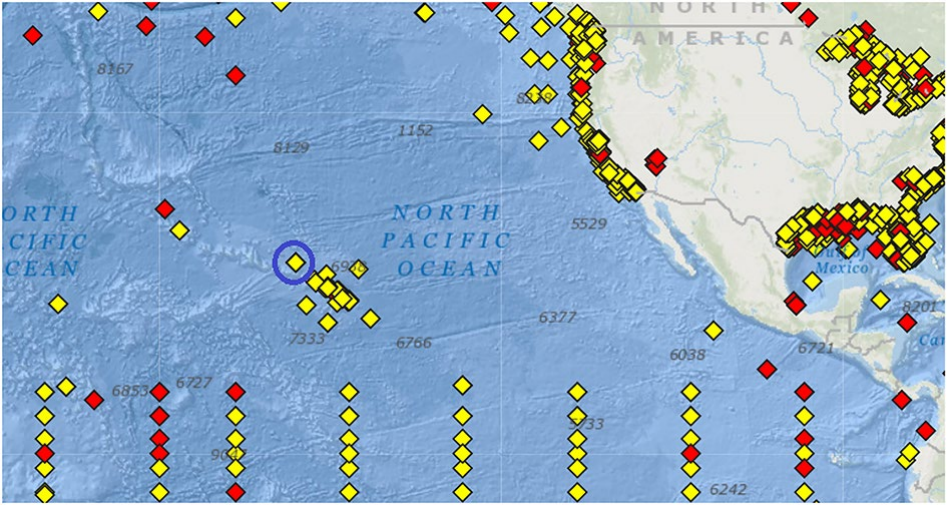
Wind and wave measurements location according to National Oceanic and Atmospheric Administration, blue circle indicates measurement station of interest. Figure taken from National Oceanic and Atmospheric Administration, https://www.ndbc.noaa.gov.

**Figure 2:**
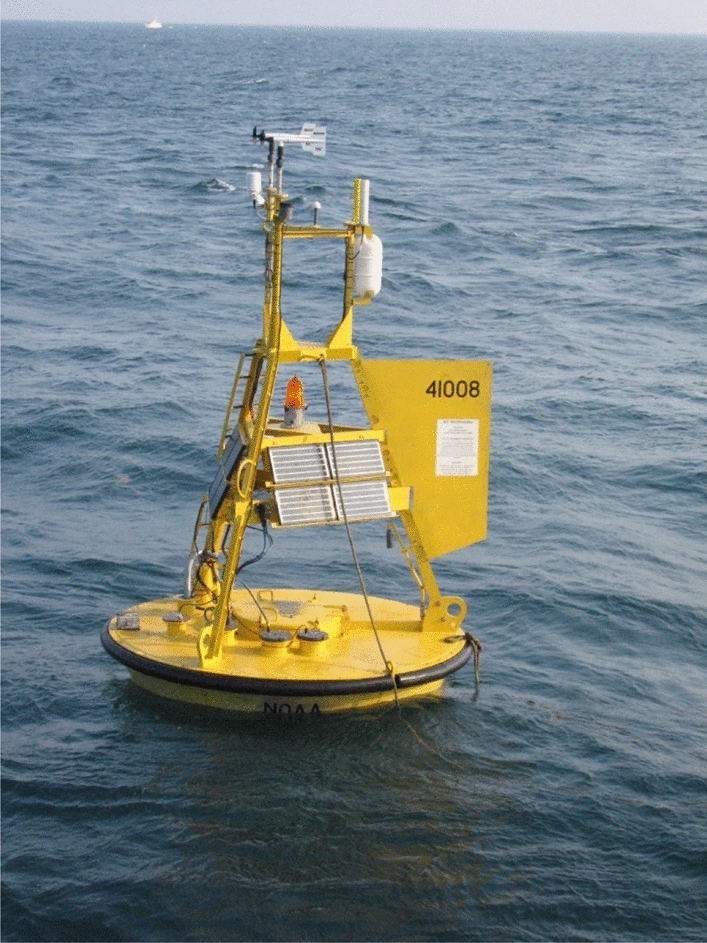
National Oceanic and Atmospheric Administration buoy^54^.

Figure 1 presents National Oceanic and Atmospheric Administration^54^, buoy locations in North Pacific, blue circle indicates area of interest. Figure 2 shows data buoy containing sensors used to monitor and collect atmospheric and oceanographic conditions, collected data is then converted into an electronic signal and transmitted to shore or logged in the onboard data unit. This 3-m foam buoy has following characteristics:

Site elevation: sea level.

Air temp height: 3.4 m above site elevation.

Anemometer height: 3.8 m above site elevation.

Barometer elevation: 2.4 m above mean sea level.

Sea temp depth: 2 m below water line.

Water depth: 1987 m.

Watch circle radius: 5004 yards.

In order to unify all two measured time series $X, Y$ the following scaling was performed:

14 $$X \to \frac{X}{{\eta_{X} }}, Y \to \frac{Y}{{\eta_{Y} }}$$

making all two responses non-dimensional and having the same failure limit equal to 1. Next, all local maxima from two measured time series were merged into one single time series by keeping them in time non-decreasing order: $\vec{R} = \left( {{\text{max}}\left\{ {X_{1} ,Y_{1} } \right\}, \ldots ,{\text{max}}\left\{ {X_{N} ,Y_{N} } \right\}} \right)$ with the whole vector $\vec{R}$ being sorted in temporally non-decreasing order of occurrence of these local maxima.

**Figure 3:**
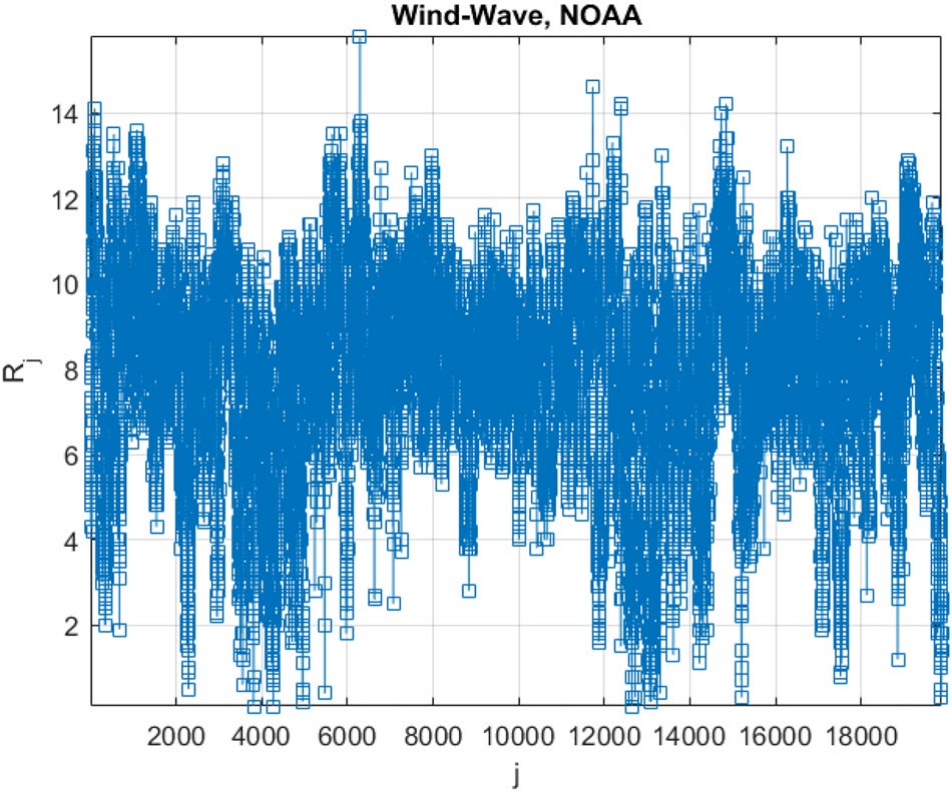
Scaled non-dimensional assembled 2D vector $\vec{\user2{R}}$.

Figure 3 presents example of non-dimensional assembled vector $\vec{R}$, consisting of assembled local maxima of raw daily largest highest daily windcast speed data. The failure probability distribution tail extrapolation was performed towards 100 years return period. Synthetic vector $\vec{R}$ does not have physical meaning on its own, as it assembled of completely different response components. Index $j$ is just a running index of local maxima encountered in non-decreasing time sequence. Index $j$ being running index of local maxima encountered in non-decreasing time sequence. The «shorter» data record was generated by taking each tenth data point from the «longer» data record.

**Figure 4:**
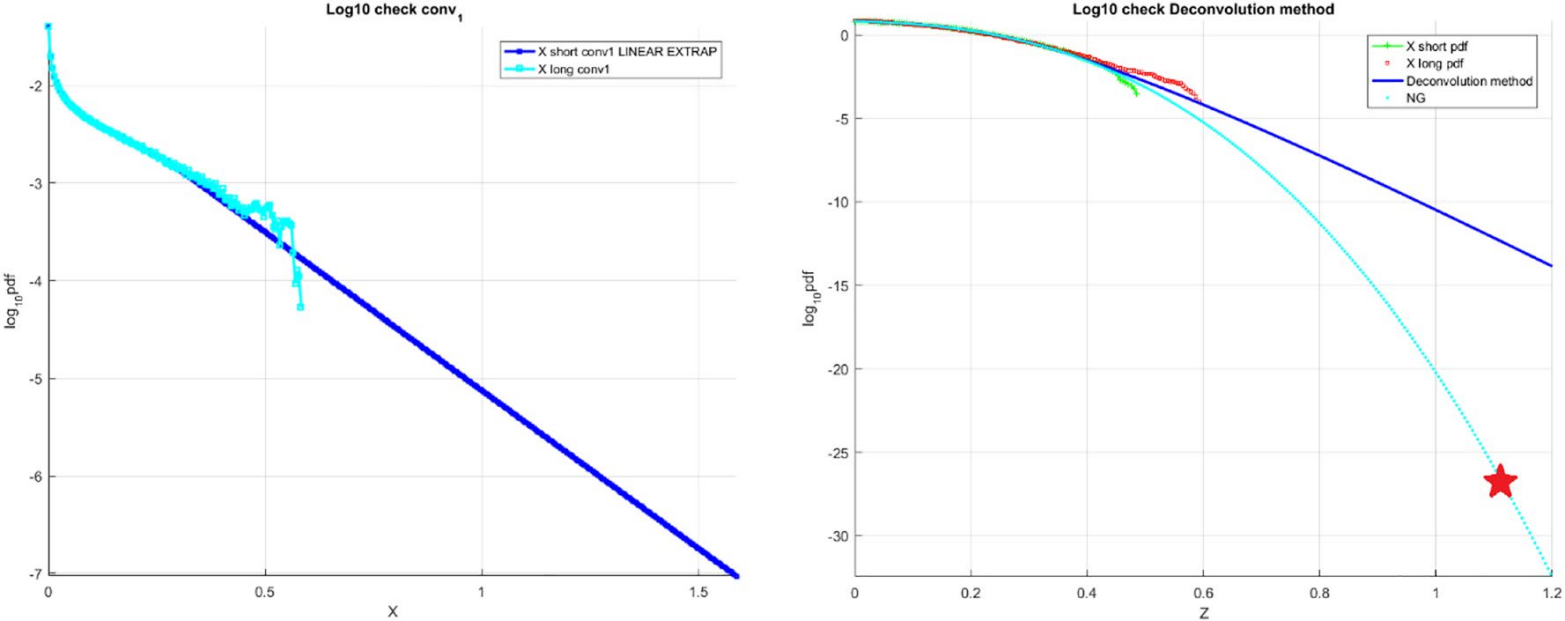
Combined wind speed and wave height data. Left: scaled $\user2{ f}_{{{\varvec{X}}_{1} }}$ tail on the decimal log scale for the «shorter» data (cyan), linearly extrapolated (dark blue). Right: unscaled raw «shorter» data (green) $\user2{ f}_{{\varvec{X}}}$ tail on the decimal log scale, extrapolated by deconvolution method (dark blue), along with «longer» raw data (red) and NG extrapolation (cyan). Star indicates 100 years return period prediction.

Figure 4 on the left presents the «shorter» data record $f_{{X_{1} }}$ tail, obtained by deconvolution as in Eq. (13), and subsequently linearly extrapolated in the terminal tail section to cover the $X_{1}$ range matching the «longer» data record. Figure 4 on the right presents final unscaled results of the proposed in this paper technique, namely the «shorter» decimal log scale $f_{X}$ tail, extrapolated by deconvolution, along with «longer» data distribution tail and NG extrapolation.

It is seen that NG prediction does not agree with deconvolution approach, leading to non-conservative prediction, indicated by star in Fig. 4 on the right. It is seen from Fig. 4 on the right that the proposed method performs quite well, being based on the «shorter» data set, and delivering probability distribution close to the one based on the «longer» data set. Figure 4 on the right presents extrapolation according to Eq. (9) as well novel deconvolution method towards failure state with 100 year return period, which is 1, and somewhat beyond, $\lambda = 0.05$ cut-on value was used. Dotted lines indicate extrapolated 95% confidence interval according to Eq. (10). According to Eq. (5) $p\left( \lambda \right)$ is directly related to the target failure probability $1 - P$ from Eq. (1). Therefore, in agreement with Eq. (5) system failure probability $1 - P \approx 1 - P_{k} \left( 1 \right)$ may be now estimated. Note that in Eq. (5) $N$ corresponds to a total number of local maxima in the unified response vector $\vec{R}$.Conditioning parameter $k = 6$ was found to be sufficient due to occurrence of convergence with respect to $k$, see Eq. (6). The generalization potential of the proposed methods is extension to non-stationary systems with an underlying trend. If the underlying trend would be known, which is rarely the case, it may be subtracted to bring measurements to stationary state. Otherwise proper trend analysis would be needed in combination with advocated here methods. The main model assumptions subsequent limitation is the system quasi-stationarity as mentioned before.

## Conclusions

The key advantage of introduced methodology is its ability to assess reliability of non-linear dynamic systems with high dimensionality.

This paper studied wind speeds and wave heights measured by National Oceanic and Atmospheric Administration buoys in North Pacific region, during years 2009–2014. Novel environmental reliability method has been applied to predict occurrence of extreme waves within time horizon of 100 years. Theoretical reasoning behind the proposed method has been given in detail.

Methods introduced in this paper, have been previously validated by application to range of dynamic environmental systems, but for only one-dimensional system responses. This study has aimed at further development of robust and simple general purpose multi-dimensional reliability method. Both measured and numerically simulated time series responses can be analyzed. In case of measured system response, as illustrated in this paper, an accurate prediction of environmental system failure probability is possible.

Finally, the suggested methodology can be used in wide range of modern engineering areas of applications. The presented environmental example does not limit applicability area of new method by any means.

## Data Availability

The datasets analyzed during the current study are available on request. Please contact Prof. Yihan Xing, email: yihan.xing@uis.no. See also^54^.
